# Stress urinary incontinence is caused predominantly by urethral support failure

**DOI:** 10.1007/s00192-021-05024-1

**Published:** 2022-01-22

**Authors:** Bo S. Bergström

**Affiliations:** 1 Department of Obstetrics & Gynecology Mora Hospital, 792 51, Mora, Sweden; 2 Overlege at Nordfjord Hospital, N-6771, Nordfjordeid, Norway

**Keywords:** Urethral pressure, Urgency, Urethral funneling, Pathophysiology, Mobility, TVT

## Abstract

Whales are mammals that can dive to depths of > 1000 m without the high water pressure pushing open their mouth or anus. The same is true for the female urethra. The meatus externus and internus are seals that cannot be pushed open by high water pressures. Recent evidence suggests that the female meatus internus is pushed open when the bladder pressure exceeds the urethral pressure. For a relaxed detrusor, this opening is not possible for at least three reasons: the law of elastic collision, Pascal’s law of hydrostatics and the Hagen-Poiseuille law. The three laws do not support that urethral function failure is the predominant cause of stress urinary incontinence (SUI); however, they do support that urethral support failure is. Influential urogynecologists claim the opposite. TVT surgery, according to the integral theory of SUI (IT), has high failure rates because it does not principally prevent the urethra from hanging on a less mobile bladder neck. In the case of a long urethra, the tape is set too distally, and in hypomobile SUI, the use of a tension-free suburethral tape is unwarranted/ineffective, because the proximal urethra is not elevated above its resting position. A successful operation corrects urethral support failure and not urethral function failure.

## Discussion

**Fig. 1:**
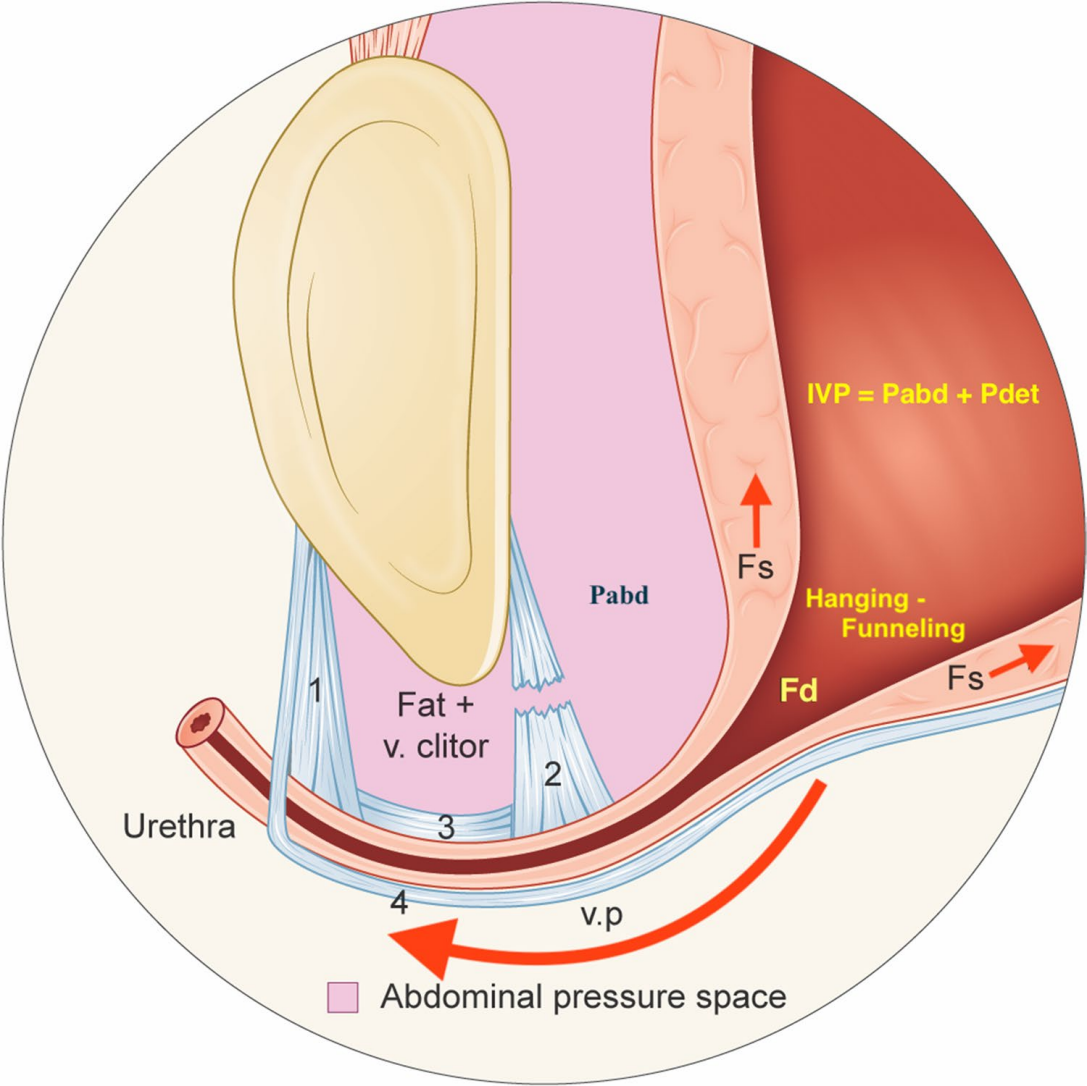
Illustration of hypermobile stress urinary incontinence during a Valsalva maneuver. In the illustrated case, the Pabd is just less than the abdominal leak point pressure (aLPP), and thus there is hanging/forced funneling without urine leakage. The maximal urethral pressure during stress (sMUP) resists the distending force (Fd) but the enforced distension of the proximal urethra may provoke urgency and frequency symptoms [4]. (1) Right anterior pubourethral ligament, which attaches to the pubocervical fascia (PCF), (2) right posterior pubourethral ligament which attaches to the PCF, (3) right intermediate pubourethral ligament, which attaches to the PCF (between this ligament and the os pubis, there is only fat and a ramus of vena clitoridis) and (4) PCF. Abbreviations: Fd: outflow distending force, Fs: pulling/ shearing force, v. clitor: ramus of vena clitoridis, v.p.: vaginal point (which corresponds to the attachment point of the posterior pubourethral ligaments (PUL) to the PCF on each side of the urethra), IVP: intravesical pressure, Pabd: intraabdominal pressure, Pdet: detrusor pressure. The illustration can alternatively be interpreted to demonstrate a urethra with minimal mobility (“fixed urethra”), exhibiting hanging/“forced funneling,” even at rest

Zacharin has claimed that SUI is a mechanical problem resulting from upper urethral support failure and that the urethra is normal [1]. His model for stress urinary incontinence equals the urethral hanging theory (UHT) [2–8], except for the new idea that proximal urethral funneling is caused by forced funneling when the urethra is pressed down to hang on a less mobile bladder neck. The vaginal point (v.p.) for making a suburethral support, corresponds to the key site of continence control stated by Zacharin: the paraurethral attachment of the two posterior pubourethral ligaments (PUL) to the vaginal wall (Fig. 1). The UHT was first described in the 1991 manual for the continence unit at Mora Hospital in Sweden (BS Bergström), but not until 2015 was the theory seen as a model for virtual stress urinary incontinence (SUI) biomechanical testing. The eight published articles on UHT are grounded on this insight.

In a recently published critical appraisal of the literature regarding female urethral function and failure [9], the authors have reviewed the ROSE study (2008), the “cough game” study (2009) and the TOMUS study (2010), among others.

The authors have concluded that urethral function failure is the predominant factor in SUI pathogenesis and that it is a hydraulic fact that the urethral closure pressure must exceed the bladder pressure for continence to be maintained.

In the ROSE study [10], urethral function was classified by measurement of the maximal urethral closure pressure at rest (MUCP), which was found to be 42% lower in the SUI group than in a symptom-free control group, with an effect size, in predicting SUI, of 1.47, explaining 50% of SUI. Lesser effect sizes were seen for support parameters. Of the four measured support parameters, “the most predictive support parameter”, the point Aa, corresponding to the urethrovesical junction/bladder neck (BN), had an effect size of 0.5, explaining 16% of SUI.

In the “cough game” study [11], ultrasound videos from the ROSE study were further evaluated in a case-control study by an expert panel, regarding the urethrovesical mobility (point Aa) during coughing. This analysis resulted in the correct identification of women with stress incontinence 57% of the time, which is only 7% better than would be expected by chance. The authors have concluded that the results confirm “that urethrovesical mobility is not strongly associated with stress incontinence.” This is true but does not exclude urethral support failure as the predominant factor for SUI.

The TOMUS study [12] has reported 1-year objective and subjective failure rates of 20% and 40%, respectively, with no difference between tension-free vaginal tape (TVT) and transobturator tape (TOT) procedures.

If Zacharin and the UHT are correct, and urethral support failure is the predominant factor for SUI, how can recent evidence show that urethral function failure is the predominant factor? The answer to this question is that the ROSE study was inaccurately designed. The authors measured several parameters but had no valid SUI biomechanical model to test. Without a correct model, it is impossible to know what to measure and how to interpret results.

The key parameter for support, urethral support in relation to BN suppport (urethral mobility in relation to BN mobility), was not measured. Pirpiris et al. have examined the correlation between segmental urethral mobility and symptoms as well as urodynamic findings. They found that SUI is strongly associated with mid-urethral mobility rather than BN mobility (point Aa). The most significant association (*P* = 0.006) was related to a point 16 mm from the BN [13]. This point corresponds to Zacharin’s “key site of continence control” [1] and to the vaginal point (v.p.) stipulated by UHT for making a suburethral support. The UHT procedure involves a suburethral tape (width = 11 mm) starting 1 cm from the BN, which is the center of tape at the v.p., 15.5 mm from the BN.

The authors of the Rose study and the cough game study should have attempted to evaluate urethral mobility in relation to BN mobility, which, according to the UHT, causes SUI. Such an evaluation would probably have shown an effect size much higher than 1.47 (MUCP). Point Aa mobility is irrelevant for causing SUI if the proximal urethra mobility is equal or lower.

Many women with a large urethrocystocele are completely continent; however, if the cystocele and the BN are reduced without correcting for the proximal urethra descent, women will be incontinent because, during stress, the proximal urethra is pressed down and hangs on the BN (de novo SUI).

Successful sling operations are known not to increase the MUCP but to decrease urethral mobility. The previously reported results [9, 10] contradict these facts; nevertheless, the authors have concluded that urethral function failure (MUCP), not urethral support failure (mobility), is the predominant cause of SUI. A 1997 statement may explain this paradoxical reasoning. DeLancey has stated that “our operations are empirical and bypass the normal continence mechanism” and that this “creates a new form of continence” [14].

The authors claim [9] that it is a hydraulic fact that urethral closure pressure must exceed the bladder pressure for continence to be maintained. However, this claim is false for the urethra, because the closed m.i. is the primary closure mechanism and is a perfect seal. The mid-urethral high pressure zone (HPZ) is a secondary closure mechanism.

The bladder pressure is independent of the conditions behind (on the other side of) a closed m.i., and, consequently, the size of the urethral pressure is irrelevant for m.i opening. The HPZ can prevent leakage of urine from the urethra but cannot prevent m.i. opening/funneling and leakage into the proximal urethra.

For a continent woman, with normal urethral support and normal spatial relationship between the proximal urethra and the BN, the MUCP can be almost zero without any leakage of urine even at high bladder pressure. SUI is also found in women with normal urethral morphology and resting pressure profiles [2]. Urethral hanging/funneling occurs only when the compliance of the proximal urethral support exceeds the compliance of the BN support.

In SUI, during stress, the proximal urethra is pressed down, so that it hangs on a less mobile BN, and a pulling force Fs shears open the m.i. together with an acutely enhanced outflow distension force, Fd (Pascal’s formula F = P × area); the proximal urethra is funneled, thus potentially affecting the HPZ. At the abdominal leak point pressure (aLPP), urine leaks from the urethra. Funneling (forced distension) at Pabd < aLPP may cause urgency with or without uncontrolled detrusor contractions, thereby explaining why most women with SUI have mixed symptoms. The urethra is subject to physical laws that cannot be broken.

The bladder-urethra complex is located inside a “water bag,” i.e., the abdominal cavity (AC), and within a pressure equalization zone, which is caudally limited by the pubocervical fascia. A Pabd increase is equally “transmitted” to the bladder and proximal urethra (Pascal’s law of hydrostatics), and as the proximal urethra remains inside the AC the abdominal pressure transmission is always 100%. The bladder pressure is perpendicular to the bladder wall and generates no pulling force able to shear open the m.i. This is per the law of elastic collision, which states that a fluid molecule bouncing against a wall generates a force perpendicular to it. When the rhabdosphincter complex and the circular smooth muscle contract in response to signals from the pontine storage center (guarding reflex), the urethral pressure but not the bladder pressure increases; the stress maximal urethral pressure (sMUP, s = stress) upsurges, and the safety margin for continence improves. The urethral lumen is zero, and its resistance to urine flow is infinite (Hagen-Poiseuille law).

Meatus internus is like an inward opening door, in that the bladder will rupture before it is opened. This aspect is consistent with the findings of a study by Bush, in which an abdominal pressure two orders of magnitude (100 times) greater was found to be be required to forcibly funnel the urethra [15]. Mammals, such as whales, can dive to depths of > 1000 m without high-water pressure pushing open their mouth or anal opening. The same is true for the female urethra. There is a zero-sum situation. The urethra remains tightly closed regardless of the bladder pressure. Muscle relaxation can decrease the urethral pressure but cannot open the urethral lumen or a closed m.i. A pulling force is necessary to open the m.i., it cannot be pushed open. Collagen, elastic fibers and the submucosal vascular plexus contribute to urethral closure. The thick submucosa and the thick relaxed longitudinal smooth muscle form an inner filler matter against which the outer structures compress. Intraluminal secretions from submucosal glands promote urethral sealing. Finally, the urethral support structures—the pubococcygeus muscles, which are connected to the posterior PUL and the pubocervical fascia (PCF)—close the urogenital hiatus and lift the anterior vaginal wall, thus pressing the posterior urethral wall against its anterior wall. This lifting mechanism maintains the normal spatial relationship between the proximal urethra and bladder neck and prevents the proximal urethra from descending to a hanging/forced funneling situation during stress. Failure of this supporting mechanism is the predominant cause of SUI. Proximal urethra descent in relation to the BN manifests as an enlarged posterior urethrovesical angle (PUVA).

Nevertheless, the same hanging mechanism and widening of the PUVA are analogous to occurrences at the start of normal micturition, thus enabling continent women to urinate by straining/pushing. The descent to hanging is then “intentional,” occurring through relaxing of the pubococcygeus muscles/PUL complex, in contrast to the “unintentional” descent caused by support failure/a defected PUL in SUI. Without this hanging mechanism that further pulls open/funnels the proximal urethra, straining/pushing would result in a “zero-sum situation” without any effect on bladder emptying because the pressure is equally increased around and inside the bladder and the proximal urethra.

During normal micturition, the m.i. is pulled open when the curved conjoined inner longitudinal smooth muscles of the bladder and urethra—that are innervated by parasympathetic nerves—contract/shorten and straighten (UHT).

Urogynecologists disagree regarding how the urethra is opened in normal micturition and in SUI. The UHT states that during stress, the suburethral vaginal wall is pressed down by the Pabd to a greater extent than the vaginal wall supporting the BN, resulting in urethral hanging on a less mobile BN. The m.i. is pulled open. Below, five other theories are discussed: Ingelman-Sundberg’s theory of a pre-tensioned pubovesical ligament (IST 1949) [16], Enhörning’s abdominal pressure transmission theory (ET 1961) [17], Petros/Ulmstens integral theory of SUI (IT 1990) [18], DeLancey’s hammock theory (HT 1994) [19] and Mostwin’s unified theory (MUT 2001) [20].

*IST:* During stress, the suburethral vaginal wall is pressed down by the Pabd, and the posterior urethral wall is sheared from the better supported anterior urethral wall. Increased tension in the pubovesical ligament (PVL) results in internal urethral sphincter insufficiency in cases of pelvic floor insufficiency. The m.i. is pulled open.

*ET:* During stress, the suburethral vaginal wall is pressed down by the Pabd without reaching a firm backstop, and the counterpressure is partially proximally relocated to the vaginal wall below the bladder, thus resulting in a damping effect in response to pressure transmission to the proximal urethra. When the bladder pressure is higher than the sMUP, the m.i. is pushed open.

*HT:* During stress, the suburethral vaginal wall is pressed down by the Pabd. The supporting tissues are unstable and do not form a firm layer against which the urethra can be compressed. Therefore, the effect of Pabd on urethral lumen transverse closure is delayed, thus allowing leakage of urine during the delay. When the bladder pressure is higher than the sMUP, the m.i. is pushed open.

*IT:* During stress, the levator plate and conjoined longitudinal anal muscles contract simultaneously, pulling the anterior vaginal wall down, and the posterior urethral wall is sheared from the better supported anterior urethral wall. IT rejects that Pabd causes SUI and that pressure transmission contributes to urethral closure. The m.i. is pulled open.

*MUT:* During stress, the suburethral vaginal wall is pressed down by the Pabd, and the posterior urethral wall is sheared from the better supported anterior urethral wall. The m.i. is pulled open.

Because ET and HT do not follow Pascal’s law of hydrostatics and the law of elastic collision, and IT rejects Pascal’s law, they cannot be considered scientifically sound.

IST, MUT and IT state that in SUI the PVL gives “better” support to the anterior urethral wall and causes proximal urethral funneling when the posterior urethral wall descends together with the anterior vaginal wall. A recent review article [21] casts doubt on the existence of a distinct PVL.

Ingelman-Sundberg assumed that SUI could be cured by dividing the PVL [16, 22]. He operated on three women; two were cured and one improved. Mulvany operated on 58 women over the course of 3 years and reported that “bladder function was restored to normalcy in all” [23]. The figure presented in the 1949 article by Ingelman-Sundberg [16] indicates that the anterior parts of the two arcus tendineus fascia pelvises were severed besides an alleged PVL.

Mulvany wrote: “A simple operation…is done by a few sweeps of the finger…the procedure, termed vesico-urethrolysis, is the reverse of the present-day type of operation which falls into the category of a vesico-urethropexy.” This is consistent with the UHT, because hanging/funneling can be prevented by increasing urethral support (preventing a urethral prolapse [urethrocele]) or decreasing BN support (creating a BN prolapse). In both cases, a normal spatial relationship is restored between the proximal urethra and BN, thereby preventing hanging. Moreover, cystocele emergence can lessen or eliminate existing SUI, and surgical overcorrection of a cystocele can cause SUI (de novo SUI).

However, although the vesico-urethrolysis procedure had a high success rate, it was never reported again, probably because of the occurrence of prolapse complications. Nevertheless, this procedure can aid in understanding the pathophysiology of SUI and is therefore referred to in this article.

The finding that MUCP is 42% lower in SUI than in women with normal continence is explained by urethral function failure covarying with urethral support failure. However, this does not implicate that urethral failure is the predominant cause of SUI. There are several explanations for this covariation.

Genetic predisposition for collagen-associated disorder results in some women being born with weaker tissues and having a higher risk for prolapse, SUI, varicose veins and other conditions. The collagen disorder involves both urethral support structures and intrinsic urethral tissues. Aging weakens muscles throughout the body. Childbirth by the vaginal route can traumatize the pelvic floor, including muscles, fascia/ligaments and nerves involved in both urethral support and urethral function. Moreover, support failures decrease the ability of the pelvic floor muscles to close the urogenital hiatus and lift the PCF, thereby resulting in less urethral pressure. Consequently, a strong covariation is observed between support defects and intrinsic urethral defects/pressure.

According to DeLancey et al. [9], “in the absence of interventions targeting low urethral closure pressure, surgical results from support operations are bound to remain imperfect” and “not addressing urethral failure” explains why success rates are only objectively 80% and subjectively 60%. These statements contradict the UHT, which states that most failed operations depend on sustained uncorrected suburethral support; a closed m.i. is a perfect seal, and low urethral pressure is irrelevant for its opening. A successful mid-urethral sling (MUS) surgery corrects urethral descent in relation to the BN but does not change the MUCP. Restoration of defective suburethral support immediately corrects urethral funneling, thus demonstrating that the cause is functional rather than morphological.

Mid-urethral sling (MUS) surgery has an objective failure rate of 10%–20%. However, women with hypomobile SUI have a higher failure rate, and many women who are cured of SUI experience de novo or persistent symptoms of urgency and frequency that, together with eventual voiding problems, explain the 40% subjective failure rate. In the Swedish register for gynecological operations, the 1-year objective failure rate is approximately 30%. Despite these high failure rates, MUS procedures are frequently described as having high success rates.

**Fig. 2:**
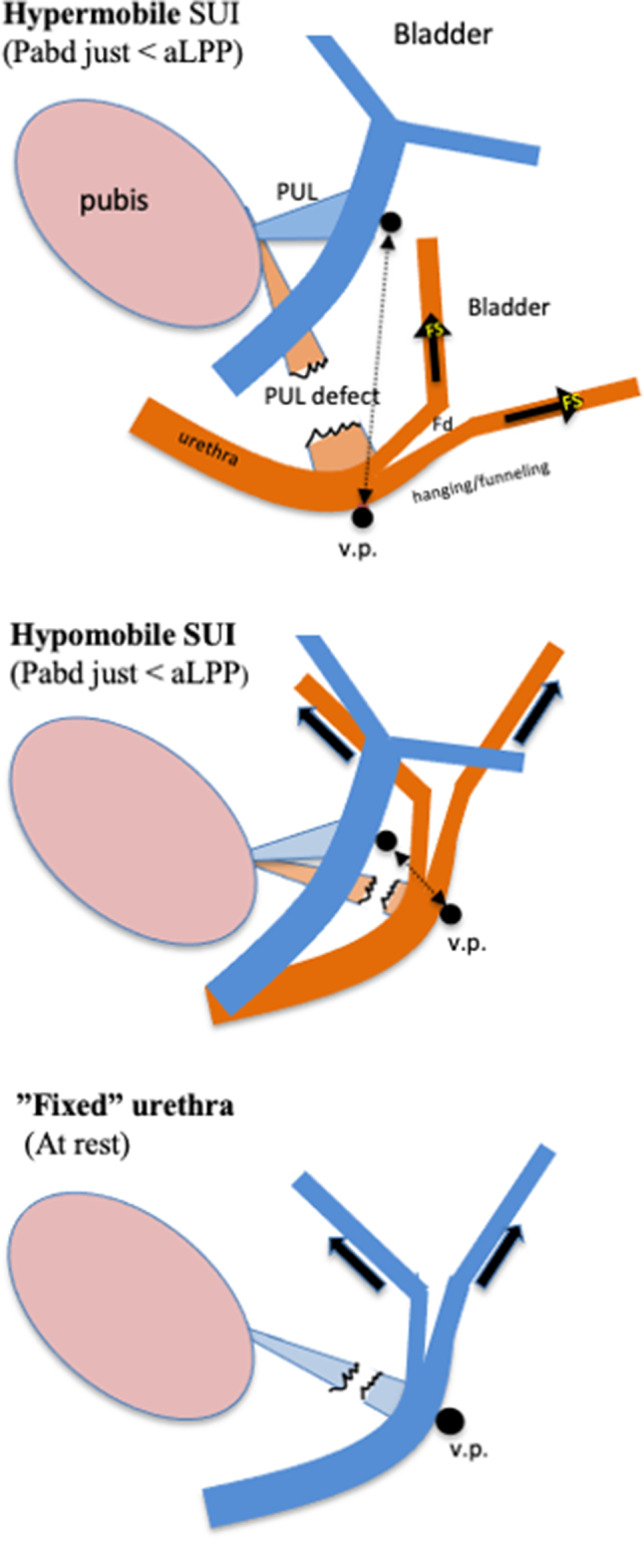
Demonstration of hanging/forced funneling in hypermobile, hypomobile and “fixed” types of SUI. It also shows the importance of the “therapeutic window” to choose between a tension-free suburethral support and a lifting support. In cases with minimally mobile BN (“fixed” urethra), i.e., exhibiting hanging/funneling even at rest, a suburethral tension-free tape is of marginal, if any, benefit to the woman. In these cases the proximal urethra at the v.p. must be lifted above its resting position. Lifting is also required in the cases with less hypomobile urethra not hanging at rest. This is because the use of tension-free vaginal tape (TVT) or transobturator tape (TOT) is associated with low cure rates as the downward distance for the urethra to reach a hanging position is short, and a high Pabd makes the TVT and TOT sway downward a little owing to their elasticity. A TOT, in particular, sways downward because it is similar to a 5–8-cm-long horizontal hammock that is laterally fixed on soft tissues. This is in contrast to a TVT, which forms a tight vertical loop that is short because it adheres to the lower part of the bony pubic body postoperatively. To create a lift without the risk of obstruction, the “TVT technique” can be employed to insert one tuned tape in the paraurethral tissue on each side of the v.p. or alternatively to elevate the proximal urethra by broadly folding the pubocervical fascia at the v.p. and then supporting the plicated fascia with a tension-free suburethral tape (TVT); the plicated fascia creates a broad cushen between the urethra and the tape that prevents obstruction problems. PUL, right posterior pubo-urethral ligament which attaches to the PCF; blue color, urethra at rest; brown color, urethra during stress; black arrow, therapeutic window (t.w.); Fs, pulling/shearing force; Fd, outflow distending force; aLPP, abdominal leak point pressure. The distance between the v.p. at rest and the v.p. at the abdominal leak point pressure is the “therapeutic window” (t.w.). A TVT located inside the t.w. is curative. The t.w. can be estimated by holding a fingertip a short distance under the v.p. at rest and asking the woman to perform a slow Valsalva maneuver. The maximum “curative” distance is the t.w. In hypermobile, hypomobile and “fixed” types of SUI, the t.w is large, small and nearly zero, respectively

Hypermobile SUI comprises 80%–90% of cases and has high cure rates, nearly independently of the tape position. According to the UHT, the 10%–20% of cases with hypomobile SUI corresponds to the 10–20% objective failure rate. According to the UHT, an immobile “fixed” urethra corresponds to hanging/funneling even at rest, when the urethra is tethered to an immobile BN, thus limiting its descent (Fig. 2).

In “fixed” cases of SUI, a suburethral tension-free tape is of marginal benefit to the woman. To prevent hanging, the proximal urethra at the vaginal point (v.p.) must be lifted above its resting position. Funneling existing even at rest cannot be treated by a tension-free tape loosely placed under the posterior urethral wall (Fig. 2). Lifting is also required in the case of a less hypomobile urethra not hanging at rest, because use of TVT or TOT is associated with low cure rates, given that the downward distance for the urethra to reach a hanging position is short, and a high Pabd makes the TVT and TOT sway downward owing to their elasticity. A horizontal 5–8-cm-long TOT laterally fixed on soft tissues sways downward more than a TVT, because a TVT forms a tight vertical loop that is short and is postoperatively adhered to the lower part of the bony pubic body. To create a lift without an obstruction risk, the TVT technique can be used to insert one tuned tape in the paraurethral tissue on each side of the v.p. or to elevate the proximal urethra by broadly folding the PCF at the v.p. and then supporting the plicated fascia with a tension-free suburethral tape (TVT).

A TVT placed starting at 1 cm from the BN implies that the tape center is positioned at the v.p. Consequently, in case of a long urethra (45 mm), the tape position is proximal, and in the case of an average long urethra (30–35 mm), the tape position is mid-urethral [4]. The v.p. corresponds to the posterior PUL attachment to the PCF on each side of the urethra, which is the key site of continence control in the female [1]. Conjecturally, a short urethra has a foreshortened extra-abdominal part; consequently, the posterior PUL attachment to the vaginal wall may be found at approximately the same distance from the BN and equally at the midpoint of the intra-abdominal urethra. A successful operation corrects proximal urethral descent in relation to the BN but does not change the MUCP.

The IT concept, described by Ulmsten in 1996, whereby the PUL acts as a fulcrum, led to the decision to recreate the PUL by setting a TVT starting 0.5 cm from the meatus externus [24]. This location was later changed to 1 cm (1999). Such distal tape positions, defined by a distance from the m.e., were probably based on Ulmsten’s 1982 study of 25 normally continent women, showing that the urethral “knee” is located 15 mm from the m.e. and the HPZ 5 mm proximal to the knee. The knee was found to represent the site of the PUL and the site where the urethra perforates the urogenital diaphragm [25].

Ulmsten has asserted that the sling must critically be placed at this location [24]. A TVT is positioned at the knee when it is set starting 1 cm from the m.e. Ulmsten’s first stipulation of a distance 0.5 cm from m.e. might be explained by an initial confusion regarding the urethra’s anatomical and functional lengths where the knee is located at 56% and 72%, respectively [25].

TVT surgery according to IT does not discriminate between hyper- and hypomobile SUI, and the procedure has high failure rates because it does not principally prevent urethral hanging. In cases with hypermobile urethra, the success rate is very high and almost independent of the tape position. However, for a long urethra, the tape is set too distally, even if set midurethrally. The SUI is cured because the urethra is compressed/kinked in its distal part but, during stress, hanging/funneling persists proximally and may cause urgency symptoms [4]. In hypomobile SUI, use of a tension-free suburethral tape is unwarranted/ineffective because the proximal urethra (v.p.) is not elevated above its resting position.

In women with hypermobile SUI (80–90% of cases) and average long urethra, the “classical” MUS procedure and the UHT procedure are exactly the same. A tape set 1 cm from the m.e. or 1 cm from the bladder neck is located at the same mid-urethral position. Thus, the difference between classical MUS surgery and “UHT surgery” is how to set the tape in cases with a long urethra and in cases with hypomobile SUI. A tape set according to UHT is always positioned at the vaginal attachment of the posterior PUL irrespective of a long or short urethra. In the classical MUS procedure, the longer the urethra, the more distally set is the tape and not at the posterior PUL attachment.

Attempting to cure hypomobile SUI without a lifting support results in high failure rates. In 2015, Volker Viereck et al. reported the outcomes of different TOT positions for various grades of urethral mobility. The cure rates for hypermobile, normomobile and hypomobile SUI, respectively, were high, low and zero, respectively [26]. The results of that study are nearly identical to those predicted in a theoretical analysis of TVT/TOT surgery through a virtual SUI biomechanical approach based on UHT [4].

Petros criticized the UHT in August 2021 [27], noting “Really a hypothesis. Bergström has never presented any experimental evidence to support his statements.” In my opinion, I am on solid ground because the SUI biomechanical model allows for meaningful comparison with thousands of good clinical studies published over the last century. I have spent considerable time searching databases for evidence that contradicts the UHT. Rather than identifying flaws and weaknesses, I have identified reports and studies that support the theory. Even a study by Petros supports the UHT. He describes a clinical experiment using two types of virtual operation techniques: a hemostat test (HT) and a pinch test (PT). HT corresponds to supporting the posterior PUL = TVT position, and PT involves a one-sided fold of the suburethral vagina [28]; such folding unavoidably results in shortening of the vaginal hammock, thereby causing a small elevation. Petros reports that up to 20% to 30% of patients require tightening of the hammock (‘pinch’) in addition to a midurethral anchoring to control urine loss on coughing when tested with ‘simulated operations’ [29]. However, Petros did not identify these women requiring a tightening/folding of the vaginal wall as women with hypomobile SUI or that vaginal folding implies a “lifting procedure.” He proposed that the folding increased the contractile activity of the horseshoe-shaped rhabdosphincter, because the folding improved its insertion points in the vaginal wall. Thus, Petros’ interpretation of the pinch maneuver is in agreement with the opinion of Delancey et al., who reported that correction for urethral function failure is necessary to avoid imperfect results. This conflicts with the UHT, according to which the urethral pressure is irrelevant for the m.i. opening, which not only corresponds to the laws of physics but also agrees with the findings of a clinical study showing that in SUI with MUCP ≤ 20 cmH_2_O, the reduced cure rate is due to a subgroup of women with a hypomobile SUI [30]. Many studies I would have liked to do are already done and support the UHT.

Two interesting clinical studies would be:

1. A study evaluating lifting support in cases with hypomobile SUI (Fig. 2).
2. A study evaluating the effect size for the vaginal point (v.p.) mobility in relation to BN (point Aa) mobility.

The UHT resolves the enigma described in the 6th edition of the International Continence Society Book (2017), stating that “Many patients with urodynamic stress incontinence show urethral mobility, though it is not yet known what it is about that mobility which permits urethral opening during stress.”

The UHT is a SUI biomechanical model—built on a new idea, reseach of others and laws of physics—that explains the pathophysiology of SUI and MUI and consequently how to repair defective anatomy. The key message from the UHT is that most failed operations result from uncorrected support failure. For hypomobile SUI cases, the proximal urethra must be elevated above its resting position. A plicated suburethal fascia at the v.p. creates a lifting support and a broad cushion between the urethra and the tape, preventing obstruction problems.

The total number of women who undergo SUI surgery is constantly increasing. A failed operation consumes the limited health care resources and is a misery for the woman. SUI procedures must have higher success rates.
